# “We’re in good hands there.” - Acceptance, barriers and facilitators of a primary care-based health coaching programme for children and adolescents with mental health problems: a qualitative study (PrimA-QuO)

**DOI:** 10.1186/s12875-020-01344-1

**Published:** 2020-12-19

**Authors:** Siona Decke, Karina Deckert, Martin Lang, Otto Laub, Verena Loidl, Lars Schwettmann, Eva Grill

**Affiliations:** 1 Institute for Medical Information Processing, Biometry and Epidemiology (IBE), Munich, Germany; 2 Pettenkofer School of Public Health, Munich, Germany; 3 BKK Vertragsarbeitsgemeinschaft Bayern, Munich, Germany; 4 Berufsverband der Kinder- und Jugendärzte (BVKJ) e.V., Cologne, Germany; 5 PaedNetz Bayern e.V., Munich, Germany; 6 Helmholtz Zentrum München (GmbH), Institute of Health Economics and Health Care Management (IGM), Neuherberg, Germany; 7 Department of Economics, Martin Luther University Halle-Wittenberg, Halle (Saale), Germany; 8 German Center for Vertigo and Balance Disorders, University Hospital, LMU Munich, Munich, Germany

**Keywords:** Mental Health Problems, Children and Adolescents, Paediatrician, Health Coaching Programme, Qualitative Study

## Abstract

**Background:**

11.5 % of girls and 17.8 % of boys are affected by a mental health problem (MHP). The most prevalent problem areas are behavioural problems (girls/boys in %: 11.9/17.9), emotional problems (9.7/8.6) and hyperactivity problems (4.8/10.8). Primary care paediatricians are the first in line to be contacted. Nevertheless, even for less severely affected patients, referral rates to specialised care are constantly high. Therefore, a major statutory health insurance fund introduced a Health Coaching (HC) programme, including a training concept for paediatricians, standardised guidelines for actions and additional payments to strengthen primary care consultation for MHP and to decrease referrals to specialised care. The aim of this study was to examine how the HC is perceived and implemented in daily practice to indicate potential strengths and challenges.

**Methods:**

During a one-year period starting in November 2017, a series of guideline-based interviews were conducted by phone with HC-developers, HC-qualified paediatricians, parents and patients (≥14 years) treated according to the HC programme. Paediatricians were selected from a Bavarian practice network with a total of 577 HC qualified paediatricians. Parents of patients with the four most common MHP diagnoses were approached by their health insurance: [World Health Organization, 2013] developmental disorder of speech and language [Wille N, et al., 2008] head/abdominal pain (somatoform) [Holling H, et al., 2003-2006 and 2009-2012] conduct disorder [Plass-Christl A, et al., 2018] non-organic enuresis. 23 paediatricians, 314 parents and 10 adolescents consented to be interviewed. Potential participants were selected based on purposeful sampling, according to principles of maximum variance. All interviews were recorded and transcribed verbatim. Two researchers analysed the transcripts independently of each other. Structuring content analysis derived from Mayring was used for analysis.

**Results:**

11 paediatricians, 3 co-developers, 22 parents and 4 adolescents were included. Families were generally satisfied with paediatric care received in the programme’s context. The HC supported paediatricians’ essential role as consultants and improved their diagnostic skills. Lack of time, financial restrictions and patients’ challenging family structures were reported as major barriers to success.

**Conclusion:**

The HC programme is perceived as a facilitator for more patient-centred care. However, structural barriers remain. Starting points for improvement are further options to strengthen families’ resources and expanded interdisciplinary networking.

**Supplementary Information:**

The online version contains supplementary material available at 10.1186/s12875-020-01344-1.

## Background

Mental health problems (MHP) of children and adolescents can considerably affect individual health and quality of life as well as performance at school and later professional development [1, 2]. Besides the risk of chronicity, there is also the risk of developing comorbidities [3, 4]. Moreover, these conditions can be of economic burden for families and healthcare systems [5–7]. Therefore, MHP are of high public health relevance in all countries of the world [1, 8]. The prevalence of MHP in Germany is high: According to the German Child and Youth Health Survey (KiGGS), around 17 % of children and adolescents aged 3-17 years are affected [9]. Among MHP, developmental disorders (17%), followed by conduct disorders (11%) are the most frequent conditions encountered in paediatric care [10]. Effective and evidence-based therapies for children and adolescents with MHP have been established, e.g. cognitive-behavioural therapy [11] or speech therapy [12]. Nevertheless, it has been reported that appropriate medical care is available to only 30 % of children and adolescents with MHP in Germany [13] and other industrialised countries [14, 15].

In Germany, primary care paediatricians are often the first in line to be consulted for MHP [13] or detect MHP during the routinely and periodically conducted developmental checks [16]. Yet, it could be shown, that the majority of primary care paediatricians does not feel adequately trained to diagnose and to treat MHP and tends to underdiagnose and undertreat MHP patients in primary care [17, 18]. Depending on the respective diagnosis, a considerable part of children with suspected MHP are subsequently referred to paediatric centres with specific mental health expertise, to speech therapists or to psychotherapy [19]. As a consequence of referral to specialised services, a number of barriers may impede or delay timely access to professional assessment and therapy. Among these barriers, waiting time, settings that fail to meet parents’ and childrens’ needs, long travelling distances and lack of intersectoral communication and treatment have been identified as the most relevant [8]. Referral rates could be decreased by interventions targeted at the primary care sector. It has been shown that one of these promising interventions, enhanced training, permits primary care physicians to detect and deliver simple interventions [20–23].. However, acceptance and perception of paediatricians and families involved have been neglected.

With the aim of providing improved integrated care for children and adolescents with MHP, a major German statutory health insurance fund (BKK-LV) in collaboration with a professional association of paediatricians (BVKJ e.V.) has introduced a programme for their insurees targeted at primary care paediatricians (Health Coaching - HC) in 2013 [24]. This includes a training concept for paediatricians, standardised guidelines for actions for 16 defined diagnostic entities, and additional fees for paediatricians who undergo this specific training and demonstrably act according to the guidelines. This approach follows the International Classification of Functioning, Disability and Health - children and youth version (ICF-CY) and was based on mutual consultations of medical stakeholders. ICF-CY is a complex classification standard that provides a common language and framework for planning and formulating support, therapy and treatment goals [25]. It takes developmental peculiarities and special living environments of children and young people into account. For example, it covers the ability of combining words into sentences, social interactions and focusing attention.

The programme has not been systematically evaluated yet. As a result, acceptance and remaining barriers to effective care within this programme still need to be captured. The objective of this qualitative study was therefore to investigate how anticipated aims of the HC are perceived and accepted by paediatricians and affect children, adolescents and their parents. These results are likely transferable to other primary programmes involving families as well. Effectiveness of the programme will be examined elsewhere in an additional quantitative study. To facilitate reading, following abbreviations are used in this manuscript:

- MHP: mental health problems
- HC: health coaching

## Methods

### Study design

In a qualitative approach we conducted a series of structured interviews with an interview guide (“guideline-based”). Paediatricians who had completed the HC training, parents of children participating in HC (< 14 years of age) and participating adolescents (≥ 14 years of age) took part.

### Setting and sample

HC is predominantly implemented in Bavaria, one of the largest federal states of Germany with a total of 13 Million inhabitants. Participating paediatricians were members of a Bavarian network of paediatricians (“PaedNetz Bayern”). Over 80% of the primary care paediatricians in Bavaria are members of PaedNetz Bayern. Currently, more than 700 members^1^ are qualified to participate in the HC programme. We included resident paediatricians in Bavaria, qualified and experienced in the HC programme. Practices that only treat private patients were excluded. In total, 23 paediatricians consented to participate. Eligible paediatricians were approached by email and selected based on purposeful sampling regarding urban/rural distribution. Table 1 gives an overview of how many participants were recruited and interviewed in each category.

**Table 1:** Overview of recruited and interviewed participants

	Paediatricians		Parents	Patients (≥ 14 Jahre)
Potential HC participants	577		565	29
Interested in an interview	23		322	10
Diagnosis is known	-		128	*-*
*Withdrawals*	*0*		*5*	*6*
Selection				
Interview feasible	**14**		**22**	**4**
	**11 PAED**	**3 DEV**		

Parents were included if at least 1 of their children had been diagnosed with 1 of the 4 most frequent MHP diagnoses indicated by the ICD code^2^ (10^th^ revision), was insured by BKK, and had been included into the programme by a HC qualified paediatrician. Parents and children were only included if they were aware of the diagnosis (self-statement). Included diagnoses were a) developmental disorder of speech and language (ICD Codes: F80.0-F80.9), b) head and abdominal pain (somatoform) (G44.2, G43.0, G43.1, F45.4, R10.4), c) conduct disorder (F68.8, F91.0-92.9, F94.0-95.9, F98.3-F98.9) and d) non-organic enuresis (F98.0). Eligible parents were invited by their health insurance by letter. We interviewed parents of children under 14 and consenting adolescents aged 14 and older. All invited participants received age-appropriate study information with the possibility to contact the study centre in case of questions. In total, 322 parents and 10 adolescents were willing to be interviewed. By the time of response, 1 adolescent had reached majority age and was therefore excluded. A total of 128 parents fulfilled our inclusion criteria. Potential participants were then selected based on purposeful sampling, according to principles of maximum variance regarding diagnosis, age, gender, social class and urban/rural distribution.

### Data protection and ethics

Approval from the Ethics Committee and the Data Protection Officer of the Medical Faculty of the Ludwig-Maximilians-Universität Munich was obtained prior to the start of the study. All study participants were informed of data protection measures and signed an informed consent form before each interview. Participation was voluntary. Paediatricians and families were offered a compensation of 30 and 40 Euro, respectively. The participants were informed about the confidentiality of the interview and their opportunity to withdraw at any time without giving any justification.

### Data collection

Interviews were conducted exclusively via telephone because of geographical distances and feasibility reasons. Since the interviews were conducted via telephone and recorded using audio devices, field notes were not necessary. The interviews were conducted by 1 researcher (SD, VL) skilled in qualitative research. Interviews were also randomly and intermittently supervised by a second researcher (SD, VL, EG, all female (female study team)), for reasons of quality control. In this sense, assumptions and attitudes, occurrences of new themes and the point of data saturation were constantly checked and discussed by the researcher involved (internal and external validity).

**Fig 1:**
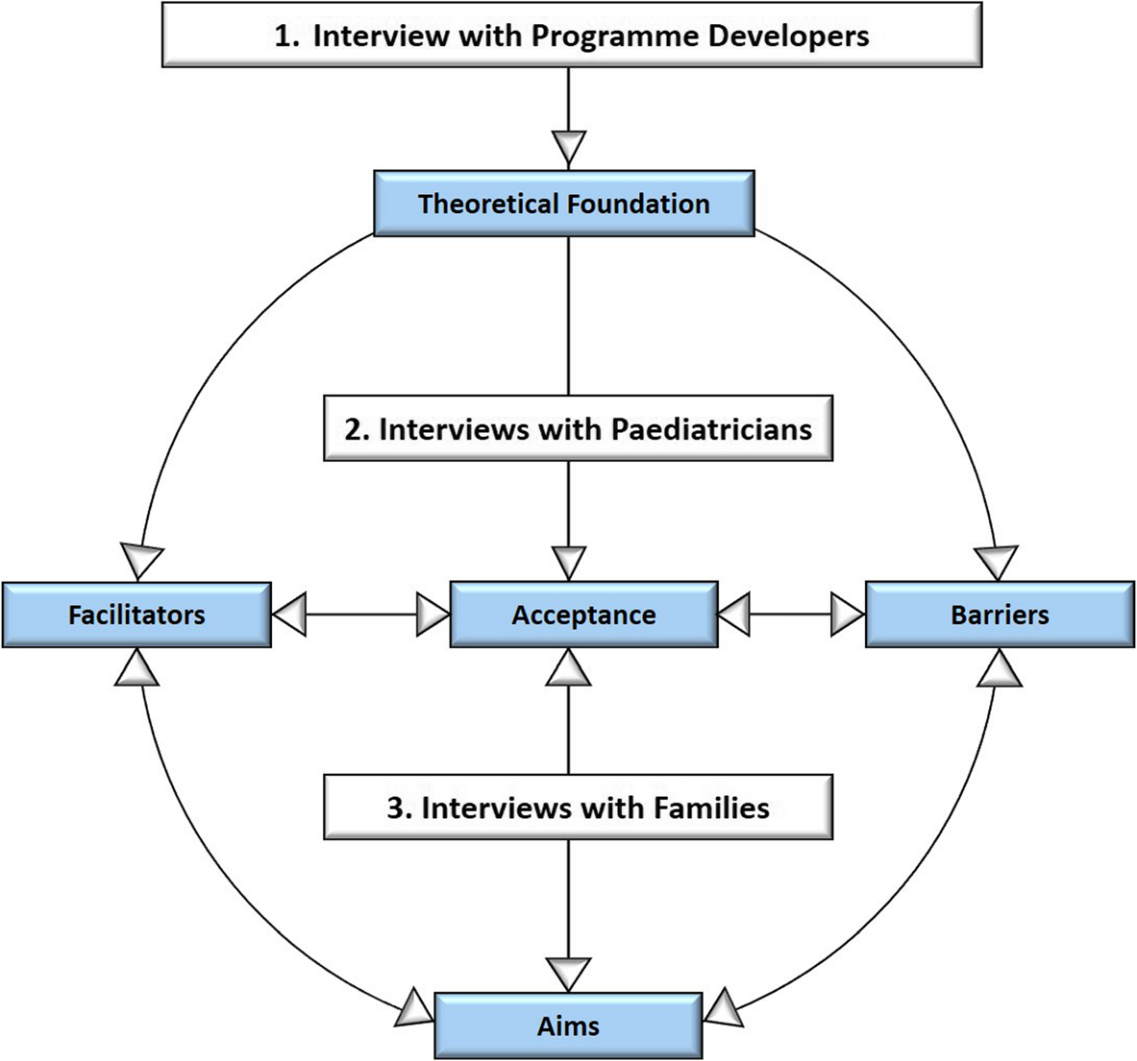
Process of data triangulation in the interviews with programme developers, paediatricians and families

Prior to the start of the actual data collection, we led an exploratory interview with the HC developer as named by PaedNetz (not shown in this publication). Problems of real-world programme implementation, facilitators and barriers of the programme and potential need for improvement were reported as most relevant issues. Based on this interview, we constructed interview guidelines for paediatricians and families. Our considerations were also substantiated by international studies assessing facilitators and barriers to manage MHP in paediatric care on the part of the doctors [17] as well as facilitators and barriers of parents seeking help for their child [27]. Following this, the guide contained questions regarding acceptability of the HC, satisfaction with MHP care in the context of the programme, quality of interaction with the paediatrician, decision making processes and shared decision making, as well as potential need for improvement. In a second step, we conducted interviews with HC qualified paediatricians incorporating their perception to further refine the interview guides for families. In this sense, we led exploratory interviews with 11 out of 21 parents before the interview guide was finalised. The guidelines were constructed according to Helfferich [28]. The structure of the interview guideline ensured that all important predetermined topics were covered, that the conversation could be guided in a targeted manner and that important topics were not forgotten. The open nature of the questions allowed expression of individual concern. Prompts and interview guides were subsequently pretested to assess understandability, phrasing and appropriateness of wording. All interviews were included in the analysis. The rigorous process of data triangulation is illustrated in Figure 1.

Interviews were audio-recorded and subsequently transcribed verbatim. Participants did not get the opportunity to review the transcripts. There were no repeat interviews. Interviewers were instructed on how to keep the conversation going by concrete inquiries of the interview guideline. In case of distress and sensitive issues, the researchers were trained to keep a friendly but professional conversation, to remain as neutral as possible and keep the focus on the topic of inquiry. Sample size was determined by saturation. The interview guides and supplementary information to the methodological approach are given in the additional file 1.

### Data analysis

Two researchers (SD, VL) analysed the transcripts independently of each other. Following the structured interview guide, a content analysis approach derived from Philipp Mayring [29, 30] was applied.

**Fig 2:**
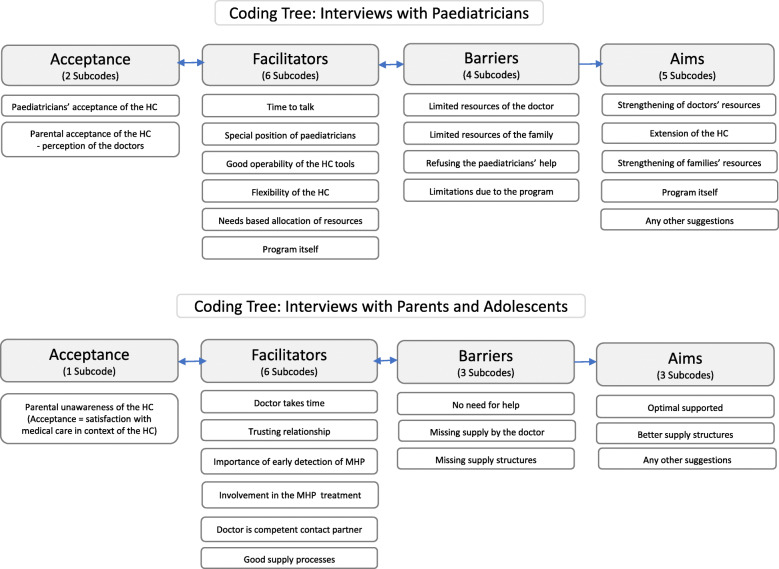
Extracted meta- and subcodes from the interviews with paediatricians, parents and adolescents

The aim of this approach is to create a category system in which each text passage is classified, and the structure of the material is recorded. This is done by defining categories, using classic examples, and coding rules. Following this approach, the material is systematically analysed by the previously developed category system. A deductive and an inductive approach to coding were chosen, which allowed to deductively allocate statements from the interviews to the various main topics (“metacodes”) of the interview guideline. Concurrently, the inductive procedure enabled the coding of the interviewees' statements within a priori defined categories while also developing new categories that had not previously been defined. Following this, the relevant text passages were systematically identified and assigned to the appropriate meta- and subcodes (Figure 2).

During the coding processes and generalisation of the material, new categories were added to the coding tree in cases where a statement could not successfully be assigned to one of the pre-specified codes (inductive approach). After having coded a small number of interviews, the coding tree was discussed among authors and adjusted accordingly. Added codes were then either differentiated or removed.

For example, the metacode “acceptance” comprises 2 subcodes in the paediatricians’ coding tree, and 1 subcode in the parental tree. Paediatricians’ acceptance of the HC was assessed, but paediatricians were also asked to describe the acceptance of the programme by the patient’s parents and the patients themselves (subcode: “Parental acceptance of the HC - perception *of the doctors”*). This was contrasted with the parental statements regarding their acceptance or rather satisfaction with medical care in the context of the HC.

Table 2 shows an extract of the parental metacode “facilitators” including the 1^st^ and 2^nd^ subcode (“doctor takes time” and “trusting relationship”) and classic interview statements within the respective category. The presentation of results is limited to this meta-level as the interviews and interview trees are too complex to show in detail.

**Table 2:** Extract of facilitators within the parental coding tree

Extract of facilitators	Subcodes	Category	Classic example
	**Doctor takes time**	extensive consultation	"That's really one of the points why we've never changed. She really takes a lot of time for us." (P11)
		doctor is there (in hard times)	"He said, 'Please come immediately' and he did not react the day after tomorrow or on Monday. " (P15)
		uncertain cases are clarified in depth	"...they are thinking about it and are catching up with the opinion of the colleague, that has often been the case" (P19)
	**Good relationship with the doctor**	doctor is person of trust	"I really trust her and her opinion." (P11)
		knowledge of the family background	"In such a situation it helps enormously that the doctor also knows about the situation of the child" (P1)
		in good hands	"I just feel comfortable with her. She already has helped me a lot in certain respects." (P16).
		empathy	"When it got emotional, she called for a helper to occupy him (her son) so that we could continue talking privately" (P20)
		likeable person	"Then I came to the doctor that was even more cordial." (P20)
		get along well with children/ability of treating children well	"The children also like to go there" (P15)

As only 4 adolescents consented to be interviewed, data saturation could not be reached in this group. Instead of developing a separate coding tree, we used the adolescents’ statements as a supplement to the parental interviews. We therefore used the same coding system that was applied to the parents’ interviews.

F4 (version 2012. Dresing & Pehl GmbH, Marburg, Germany, http://www.audiotranskription.de/) was used for transcription, MAXQDA 18 (VERBI Software; Consult, Sozialforschung GmbH, Berlin, Germany) was used for coding and analysis.

COREQ (COnsolidated criteria for REporting Qualitative research) checklist was used to support the complete and transparent reporting of our research. The complete checklist is provided (see additional file 2).

## Results

From November 2017 to November 2018, 14 paediatricians, 22 parents and 4 adolescents were interviewed. They were randomly selected from a total of 23 consenting paediatricians, 322 consenting parents and 10 adolescents, until saturation was reached. 5 parents who had initially given their consent and were randomly selected, withdrew their participation once they were contacted. Reasons were lack of motivation or time. All consenting adolescents were contacted and 6 withdrew. In 2 cases, their parents agreed to be interviewed instead. All randomly selected paediatricians participated as shown in Table 3.

**Table 3:** Demographic characteristic of paediatricians, parents and adolescents

Demographic Characteristics	Paediatricians (***n***=14)	Parents (***n***=22)	Adolescents (***n***=4)
Gender (female /male)	3/11	19/3	2/2
Age range in years	n.a.	32-49	14-17
Age of the child (<14 years) Mean (range)	n.a.	7.1 (3-14)	n.a.
Diagnosis of the child / adolescent			
- Head and somatoform abdominal pain	n.a.	3	1
- Developmental disorder of speech & language		5	-
- Non-organic enuresis		4	-
- Conduct disorder		5	1
- Combination of two		5	-
- None of them / do not know		-	2
Highest educational qualification	n.a.		n.a.
- University degree		6	
Higher education entrance qual.		5	
- Intermediate secondary school		6	
- Secondary school		4	
- Other		1	
School currently attended by children			
- University of applied sciences	n.a.	n.a.	1
- Intermediate secondary school			3
Migration background	n.a.	5	0
Population size of place of residence			
Major city (> 100,000)	8	7	4
Medium sized town (>20,000)	4	4	-
Small town (> 5,000)	2	8	-
Country town (≤ 5,000)	-	2	-
n.a.	-	1	-

### Description of study participants

11 of the 14 interviewed paediatricians were male. 3 paediatricians stated that they had also been involved in contract negotiations regarding HC and in HC development. 8 practices were located in a major city, 6 were located in a small or medium-sized town. Duration of interviews was 11 minutes on average (range: 5-23). The characteristics of the interviewees are shown in Table 3.

Regarding parents, a total of 19 mothers (age range 32-49) and 3 fathers (age range 39-47) were interviewed. Number of children per family ranged from 1 to 3. In 5 families, the child had a migration background.^3^ The majority of the families (n=12) were inhabitants of a small (> 5.000) or medium-sized town (> 20.000). 7 families were residents of a major city (> 100.000 inhabitants). Average duration of interviews with parents was 18 minutes (range: 4-46), with adolescents 13 minutes (range: 3-17).

### Metacodes and subcodes

Based on the results of the exploratory interviews prior to the start of the actual data collection, we defined 4 metacodes “acceptance”, “facilitators”, “barriers” and “aims”. For these, we defined 17 subcodes for paediatricians and 13 subcodes for parents/adolescents. All metacodes and subcodes are shown in Figure 2. The following short forms are used: “DEV” (HC co-developers), “PAED” (paediatricians), “PAR” (parents) and “ADOL” (adolescents) to facilitate reading.

### Metacode “Acceptance”

All DEV stated that the aim of the programme was to facilitate diagnosis and treatment of MPH in primary paediatric care to reduce the need for referrals. Separate from some rejection and indifference in the beginning among their ranks, this intention was generally well received among colleagues. PAED perceived their own competence for children with MHP as improved.

I simply can’t imagine general routine work without it anymore. (Transcription of Interview partner D5 (DEV), p. 1, lines 14-20)

PAED had the impression that PAR did not care about being in a specific programme but appreciated their increased efforts.

They are happy when the medical conditions we are dealing here with (…) can be treated in the practice of their confidence on an outpatient and on-site basis. (Transcription of Interview partner D5 (DEV), p. 3, lines 86-91)

Accordingly, PAR and ADOL reported that they did not realise that there was a programme specifically designed for their needs.

### Metacode “Facilitators”

DEV stated that flexibility and operability of the programme were planned and implemented right from the start. PAED appreciated the additional allocation of resources as a token of trust and valuation. Repeatedly, PAED reported that the material provided by HC was helpful and facilitated diagnosis and decision-making. They also valued the continuing training opportunities.

It allows and structures the approach, in the diagnostics itself in the practice, but also for the diagnosis and, finally, in the decision whether we want and we are able to continue the treatment in the practice. (Transcription of Interview partner D14 (PAED), p. 1, lines 12-16)

PAR and ADOL felt that their PAED allocated a large part of his consultation time to their problems. This was perceived as an indication of high quality of care. PAR mostly reported that they trusted their PAED even with more sensitive issues. Quality of communication and an inclusion in the process of decision-making were appreciated.

I am very satisfied, got a lot of advice and I think, if I go there again now and say: "Well, it has not worked yet", I will be well advised again. At the moment, I cannot think of a better way. (Transcription of Interview partner P13 (PAR), p. 9, lines 348-351)

Interaction between care providers, e.g. good connections between PAED and speech therapists, was positively noted.

### Metacode “Barriers”

Although substantial financial resources were allocated through the programme, PAED still perceived their opportunities for interaction with the patients as limited. Time and budgetary restrictions were still reported as major barriers to success. Due to the large amount of managed care contracts PAED also reported feeling overwhelmed.

DEV admitted that a realistic resource estimate should be made before the enrolment of a patient into the HC. PAED reported that it was still difficult to refer patients, and that parents might have problems to follow-up on that referral.

We detect children with MHP, but it still takes far too long until they receive therapy. (Transcription of Interview partner D13 (DEV), p. 6, lines 205-213)

PAED perceived distinct social disparities, and cultural and linguistic barriers which could not be resolved by the programme. Also, they felt that parents would not necessarily trust their expertise for sensitive issues in MHP.

Some PAR reported feeling reluctant about contacting a physician for MHP of their children, either because this might be too trivial for the paediatrician, or because more specialised help would be needed. However, PAR also reported that they had delayed consulting the PAED because they had underestimated the problems.

Because of such small things like abdominal pain I do not go to the doctor." (Transcription of Interview partner P16 (parent), p. 4, lines 160-161)

PAR explained this by their impression that the PAED seemed to be stressed and in a rush. In this vein, PAED were perceived as hardly encouraging and not participative in treatment decisions, withholding treatment options or disregarding parents’ concerns. One mother felt that she was not sufficiently empowered to support her child’s therapy more actively.

When you have a problem, you need to convince the doctors to support you. (Transcription of Interview partner P14 (PAR), p. 1, lines 16-17)

PAR reported problems with secondary and tertiary care, namely long waiting lists for specialist appointments, long distances to the next specialised clinic, limited prescription options of the PAED, and a general lack of insurance coverage for many treatment options. Recommendations were perceived as not compatible with the daily life of a family.

### Metacode “Aims”

DEV and PAED underlined the need for improved interdisciplinary networking. DEV also mentioned conflicts with specialist care providers and proposed establishing mandatory care pathways. Several detailed recommendations for programme improvement were made, e.g., to facilitate prescription, and to add options to directly strengthen the resources of families (e.g. assistant at home, language support).

PAED still proposed higher reimbursement of their services and improved quality control of HC. Universal coverage by all statutory health insurance funds was mentioned.

Our goal and our hope are that at some time all insurances will take over this service and will also take for granted that they are responsible for MHP. (Transcription of Interview partner D5 (DEV), p. 6, lines 207-210)

Generally, PAR/ADOL felt sufficiently supported. However, PAR proposed to improve access to services, e.g. by allowing telephone consultations, a better communication between providers, and a more convenient localisation of specialised services.

Specialists for both of these topics are spread quite widely over the country. You really need a connection on site and if there were more cooperation with the paediatricians, that would be great. (Transcription of Interview partner P17 (PAR), p. 11, lines 326-333)

PAR proposed to involve other health professionals such as midwives and alternative practitioners in the programme. Opening treatment options e.g. including homeopathy, and financial aids were additionally mentioned.

## Discussion

This qualitative evaluation of a primary care-based programme for children and adolescents with mental health problems revealed high appreciation and acceptance of the programme among paediatricians and families. Adolescents and parents were generally satisfied with the care provided although they did not realise that the programme was specifically targeted at their needs. Furthermore, they mentioned barriers and opportunities for improvement.

Our results are in line with the international literature reporting a good applicability of structured MHP programmes in paediatric care with? increasing screening rates and treatment of MHP in primary care settings but reports for Germany remain scarce [20–23, 32]. However, paediatricians’ potential for early detection of MHP in primary care is well documented, based on a high participation rate and acceptance of primary preventive medical examination in children and adolescents [16, 33]. In the Netherlands, politicians have been promoting MHP treatment within primary healthcare for several years now [20, 34]. Almost all Dutch residents are registered with a general practitioner (GP) and the majority of children and adolescents visit their GP at least once a year. The structure of the Dutch Project ‘Eureka’ is quite comparable to the HC programme: GPs receive a lump-sum for the comprehensive assessment of children presumed to have a MHP, as well as any further treatment of the MHP in primary care. In addition, cooperation between primary and secondary mental healthcare was stimulated, leading to an increase in the provision of social workers and primary care psychologists. As a result, GPs in the intervention group were able to identify more emotional and behavioural problems than GPs in the control practices and were more reluctant to prescribe psychopharmacological medication to children. Referral rates to mental healthcare remained relatively steady, but the referrals switched from specialised to primary mental healthcare. However, feedback of the patients and parents was not included. The question whether the improved screening leads to improved access to care and improved outcomes was not addressed either.

Parents and adolescents in our study reported satisfaction with the care provided and with the involvement in treatment decisions. As parents are the gatekeepers to seeking help for their child, parental perception of barriers and facilitators to MHP treatment access are paramount. In literature, systemic and structural issues, views and attitudes towards services and treatment, the knowledge and understanding of MHP and the help-seeking process as well as the family circumstances were found to be crucial determinants for parents’ decision to seek help [27]. This is in line with the observations expressed by paediatricians in our study. It indicates that the HC programme should focus more intensely on these barriers. Our interviewed paediatricians made several suggestions to address these barriers (e.g. assistant at home). In addition, enhancing parental awareness of MHP and their perception of paediatricians’ expertise might reduce the risk of delayed therapy.

A recent meta-analysis found that a lack of providers and resources, extensive waiting lists, and financial restrictions were major barriers for successful MHP management in children and adolescents [17]. Quite similarly, paediatricians in our study complained about time and budgetary restrictions. This was unanticipated given that the HC programme was specifically designed to facilitate access by bringing more resources into the system. Increasing funding for billable services will not necessarily be part of the solution. In contrast, it might be important to strengthen and to expand formal and informal networks as well as systematically and officially include allied health professionals into structured care pathways as shown in the Eureka project. For instance, a model programme in Baden Württemberg, Germany, successfully implemented inter-professional quality circles that subsequently increased collaboration and networking [35], or collaborative nurse-led self-management support for primary care patients [36].

Our study is the first evaluation of a primary care-based programme for children and adolescents with mental health problems reflecting the patients´, parents’ and paediatricians´ perspectives. Our approach enables greater depth to the application of the programme. We also want to identify related barriers, facilitators and need for improvement in the treatment of children and adolescents with MHP. These findings will also complement the results of the cohort study among 800 patients with MHP on effectiveness and utilisation of the HC programme (currently examined elsewhere). By integrating the professional experiences of the paediatricians and families involved, further optimisation of the programme can be achieved. Furthermore, we believe our results are most likely to be applicable and transferable to other programmes involving paediatricians and families in primary care with the aim of providing optimal care and support to patients and their parents.

The main strength of our study lies in the naturalistic approach and the openness of all interviewees. Despite the sensitive topic, we felt that participants did not hold back their opinions and were eager to talk about their experiences. The inclusion of three co-developers of the HC among the interviewed paediatricians enriched our findings, too, enabling the distinction of the HC’s anticipated goals as compared to its feasibility in everyday practice.

Nevertheless, this study has some limitations. As for all studies relying on qualitative research, interviews are always at least to some degree subject to the assumptions and attitudes of the researchers involved. Thus, the assumptions were repeatedly critically examined with respect to the methodological approach and the interpretation of results. However, we are confident that the qualification of interviewers and coders has minimised this potential bias.

HC covers 16 MHP, but the evaluation of the HC programme initially focused on four selected indications. Arguably, these are the four most common MHP showing up in paediatric practices and responding well to the HC, as specified by paediatricians of PaedNetz Bavaria, but this evaluation needs to be extended. The HC is currently limited to persons insured at the BKK funds (condition: enrolled in the programme “BKK STARKE KIDS”). Since the BKK is one of the larger statutory health insurance funds with 10.9 (Bavaria: 2.4) of a total of 73.0 million insured persons in Germany^4^ [37], the results of our study are most likely to be generalisable for Germany. Furthermore, as shown in the International context, primary care programmes like the HC can be integrated into different health system structures [20, 21].

When interpreting the study results, there is a risk of attributing the described differences to the implementation of the HC. It has to be noted that physicians’ personal commitment will still be a major driver of positive experiences and high satisfaction of the families.

## Conclusion

Primary care paediatricians are providing low-threshold care and have decisive potential in the care of children and adolescents with MHP. The HC programme currently focuses on paediatricians’ resources. Our study showed several strengths but also shortcomings of this approach. A promising future direction would be to involve all necessary care providers to avoid referral bottlenecks. Furthermore, inclusion of parents and their children in decision-making should be expanded.

## Supplementary Information

**Additional file 1:.** Contains the interview guides, in- and exclusion criteria of study participants and additional information to the methodological approach of Mayring.

**Additional file 2:.** Contains the completed COREQ (COnsolidated criteria for REporting Qualitative research) Checklist.

## Data Availability

Due to the nature of this research, participants of this study did not agree for their data to be shared publicly and as a result supporting data is not available. MAXQDA 18 (VERBI Software; Consult, Sozialforschung GmbH, Berlin, Germany) was used for coding and analysis. The coding system can be provided on demand.

## Abbreviations

MHP: Mental health problems
HC: Health coaching
DEV: HC co-developers
PAED: Paediatricians
PAR: Parents
ADOL: Adolescents

## Footnotes

1 Status as of 1^st^ March 2018

2 The International List of Causes of Death (ICD) is a diagnostic classification standard for clinical and research purposes: “ICD defines the universe of diseases, disorders, injuries and other related health conditions, listed in a comprehensive, hierarchical fashion” [26].

3 Migration background was defined based on information about the country of birth of the child and the country of birth and nationality of the parents. In this sense, children and adolescents who had immigrated from another country and had at least 1 parent not born in Germany and/or of non-German citizenship had a migration background [31].

4 Status as of 1^st^ July 2019
