# Supplementary material for: “We’re in good hands there.” - Acceptance, barriers and facilitators of a primary care-based health coaching programme for children and adolescents with mental health problems: a qualitative study (PrimA-QuO)
Source: BMC Fam Pract. 2020 Dec 19;21:273. doi: 10.1186/s12875-020-01344-1 (PMC7749989; doi:10.1186/s12875-020-01344-1)
Supplement: Supplementary file 1 — Additional file 1:. Contains the interview guides, in- and exclusion criteria of study participants and additional information to the methodological approach of Mayring. [file 12875_2020_1344_MOESM1_ESM.pdf]

Additional File 1: Supplementary Information

to

"We're in good hands there." - Acceptance, barriers and facilitators of a primary care-based health coaching programme for children with mental health problems: a qualitative study (PrimA-QuO)

Siona Decke[1,2*], Karina Deckert[3], Martin Lang[4,5], Otto Laub[5], Verena Loidl[1,2], Lars Schwettmann[6,7], Eva Grill[1,8*]

[1]Institute for Medical Information Processing, Biometry and Epidemiology - IBE, LMU Munich, Munich, Germany

[2]Pettenkofer School of Public Health, Munich, Germany

[3]BKK Vertragsarbeitsgemeinschaft Bayern, Munich, Germany

[4]Berufsverband der Kinder- und Jugendärzte (BVKJ) e.V., Cologne, Germany

[5]PaedNetz Bayern e.V., Munich, Germany

[6]Helmholtz Zentrum München (GmbH), Institute of Health Economics and Health Care Management (IGM), Neuherberg, Germany

[7]Department of Economics, Martin Luther University Halle-Wittenberg, Halle (Saale), Germany

[8]German Centre for Vertigo and Balance Disorders, University Hospital, LMU Munich, Munich, Germany

*Corresponding author

Siona Decke, MPH

Postal address: Institute for Medical Information Processing, Biometry and Epidemiology - IBE

Marchioninistr. 15, 81377 Munich, Germany

Fon: +49 (0)89 / 440077483

Email: siona.decke@med.uni-muenchen.de

Contents

Appendix A: Inclusion and exclusion criteria of study participants 2
Appendix B: Interview guidelines 3
Appendix C: Methods appendix 6

Appendix A: Inclusion and exclusion criteria of study participants

A) Children/adolescents with mental health problems and their parents

Inclusion criteria:

- children aged 0-17 years insured by BKK and enrolled in the programme "BKK STARKE KIDS" and their parents
- Children had been diagnosed at least with one of the four most frequent MHP diagnoses:
  - developmental disorder of speech and language (ICD-10: F80.0-F80.9);
  - head and abdominal pain (somatoform) (ICD-10: G44.2, G43.0, G43.1, R10.4, F45.4);
  - conduct disorder (ICD-10: F68.8, F91.0-92.9, F94.0-95.9, F98.3-F98.9)
  - nonorganic enuresis (ICD-10: F98.0);
- Last paediatrician visit less than six months ago
- Attending paediatrician was qualified in HC and offered the BKK HC programme to the child
- Signed informed consent for children aged six years and older
- Signed informed consent from parents

General exclusion criteria:

- Tentative diagnosis of MHP
- Insurance gap >30 days

B) Paediatricians

Inclusion criteria

- Resident paediatrician in Bavaria
- Written consent and invitation to participate accepted
- Qualified to participate in the HC programme

Exclusion criteria

- Practices that treat private patients only

Appendix B: Interview guidelines

A) Interview guideline for paediatricians

1. First, could you tell us when and how you became aware of the BKK health coaching programme?
2. Do you notice any changes in comparison to a standard treatment?
3. In your opinion, how work-intensive is the implementation of the programme?
4. Could you describe the acceptance of the programme by the patient's parents and the patients themselves?
5. To which extent are the general principles participation, patient orientation and strengthening of existing resources fulfilled by the programme? What do you think?
6. Is there anything you particularly like about the programme? If so, what is it?
7. Is there anything that bothers? Where do you see need for improvement?
8. Where do you believe that additional support concerning the care of the patients and their parents is required?
9. Apart from that, is there anything else that is particularly important for you concerning patient care that you would like to address?

optional: additional questions

B) Interview guideline for parents of patients

Part 1 (getting started): Relationship with the paediatrician

1. First, could you briefly tell us how you heard about your child's paediatrician?
2. How would you describe the relationship with your paediatrician?

Part 2: Health Coaching experiences in the doctor's office

3. In our study, we focus on four diagnoses. These are (1) head and abdominal pain, (2) conduct disorder, (3) enuresis and (4) developmental disorder of speech and language. When you think of your child's last visit to the doctor due to any one of these diagnoses, could you describe how you experienced this visit?
4. How would you describe the health development of your child throughout the last year?

Part 3: Health Coaching principles: participation, patient orientation and strengthening of existing resources

5. As parents, would you like to be involved in the treatment of your child, and if so, how do you feel about the conversations with your paediatrician?

6. To what extent are you involved in the treatment of your child and the decisions made by your paediatrician?
7. Have you received any information material from your paediatrician that either you or your child found helpful? If so, what did you receive?

Part 4: Theoretical knowledge about the Health Coaching programme

8. Maybe you know that your child is treated according to the BKK Health coaching programme of your health insurance. Can you tell us what you know about the programme?

Part 5 (ending): points for improvement

9. Where do you see additional need for support concerning the care of your child? What do you think could be improved?
10. Apart from that, is there anything else that is particularly important for you, when it comes to your child's care, that you would like to talk about?

optional: additional questions

c) Interview guideline for Adolescents (≥ 14 years)

Part 1 (getting started): Relationship with the paediatrician

1. When you answered the questionnaire, you may have noticed that we are referring to 4 complaints. These are headache and abdominal pain, difficulties in social behavior, bedwetting and language problems. When you think about your last visit to your paediatrician, could you tell us what you found memorable from this visit?
2. How do you feel when you are at your doctor's office?
3. Can you describe how you get along with your paediatrician?
4. Concerning your health: How do you realize that you are feeling better or worse?

Part 2: Health Coaching principles: participation, patient orientation and strengthening of existing resources

5. As an adolescent/young adult, would you like to participate and decide what is happening to you at the doctor's office?
6. Can you describe how you and your doctor are talking to each other?
7. Can you describe how your paediatrician involves you and your parents in the treatment?
8. Did the doctor give you any materials or tell you something that helps you to handle your problems better?

Part 3: Theoretical knowledge about the Health Coaching programme

9. Maybe you know that you are treated according to the BKK health coaching programme. That's a programme from your health insurance. What do you know about this programme?

Part 4 (ending): points for improvement

10. What else do you want/ wish from your paediatrician or from others?
11. Is there anything else you would like to talk about that we haven't discussed yet?

Optional: Additional questions

Appendix C: Methods appendix

Structuring content analysis by Mayring

The process model of qualitative content analysis according to Mayering [1,2] comprises of nine steps (ESM_Fig. 1).

Structuring content analysis by Mayring
1. Determination of the material
2. Analysis of the emergence situation
3. Formal characteristics of the material
4. Determine the direction of the analysis
5. Theoretical differentiation of the question
6. Determination of the analysis techniques, definition of the concrete process model
7. Definition of the analysis units
8. Analysis steps using the category system (Abstract/ Explication/Structuring) and review of the category system of theory and material
9. Interpretation of the results in the direction of the question and application of content-analytical quality criteria

***ESM_Fig. 1**: Structuring content analysis by Mayering*

After description of the starting material follows the elaboration of the analysis question (step 1 and 2). This was followed by the definition of the analysis technique, the determination of the process model and the determination of the analysis units (step 4-7). The analysis should provide information on the acceptance, barriers, funding factors and potential for improvement of the HC programme. In addition, the question was how the demands of patients and parents of the medical care of the child and the actual offer match. The interviews were segregated into distinct manageable units ('meaning units'), which were subsequently defined. Meaning units are text passages which relate to one topic, enabling the creation of a coding guideline with meta- and subcodes. The coding tree with the metacodes 'acceptance', 'facilitators', 'barriers' and 'aims' for the HC programme and subcodes were created based on theoretical considerations before starting the analysis. For instance, a 'trusting relationship with the paediatrician' and 'communication at eye level and participation during treatment' are examples of subcodes within the metacode 'facilitators'. Subsequently, the subcodes were arranged according to their content within the predefined metacodes. The category system was applied and revised based on the material (step 8). The meaning units were systematically identified by two independent researchers (first and second author) and assigned to the appropriate meta- and subcodes. Concrete passages that fall under one category and are considered as typical examples of this category are cited as so-called 'anchor examples'. Coding rules were set where demarcation issues between codes arose to allow clear mapping. In the course of the coding processes and generalization of the material, new categories were added in the coding tree in cases where a meaning could not be successfully assigned to one of the pre-specified codes (inductive approach). In a continuous process,

the coding guide was refined by differentiating the added codes in a more meaningful way or by removing them.

References

1. Mayring P (2004) Qualitative content analysis. In: Kardorff E, Steinke I, Flick U (eds) A companion to qualitative research. SAGE Publication, London, pp 266–269

2. Mayring P (2000) Qualitative Inhaltsanalyse. Grundlagen und Techniken. 7 edn. Deutscher Studien Verlag, Weinheim
